# Investigating catastrophic health expenditure among people living with HIV and AIDS in South Western Nigeria

**DOI:** 10.1002/hcs2.77

**Published:** 2023-12-11

**Authors:** Adeyinka Adeniran, Omobola Y. Ojo, Florence C. Chieme, Yeside Shogbamimu, Helen O. Olowofeso, Imane Sidibé, Oladipupo Fisher, Monsurat Adeleke

**Affiliations:** ^1^ Department of Community Health and Primary Healthcare Lagos State University College of Medicine Lagos Nigeria; ^2^ Department of Community Health and Primary Healthcare Lagos State University Teaching Hospital Lagos Nigeria; ^3^ Department of Community Medicine and Primary Care, Faculty of Public Health Federal Medical Centre Abeokuta Nigeria; ^4^ Petra Global Research Centre Lagos Nigeria; ^5^ Lagos State Ministry of Health Lagos Nigeria; ^6^ International Association of Providers of AIDS Care Washington District of Columbia USA; ^7^ Fast‐Track Cities Institute Washington District of Columbia USA; ^8^ Lagos State AIDS Control Agency Lagos Nigeria

**Keywords:** CHE, financial burden, HIV care, PLHIV, Lagos State, Nigeria

## Abstract

**Background:**

This study aimed to determine the catastrophic healthcare expenditure (CHE) among people living with HIV (PLHIV) in Lagos and to identify factors associated with CHE among them.

**Methods:**

The study was a descriptive cross‐sectional survey conducted between January and March 2021 among 578 PLHIVs drawn from various healthcare facilities in Lagos where HIV care and treatment services should be provided free of charge. Data were collected through pretested questionnaires and analyzed using Stata SE 12.

**Results:**

The mean monthly expenditure on food was N29,282 ($53.2), while expenditure on healthcare averaged N8364 ($15.2). Nearly 60% of respondents experienced CHE, while around 30% had to borrow money to pay for some aspect of their medical treatment. Almost all (96%) had no health insurance plan. Respondents' group, personal income, perception of current health status, and the number of people in their households were significantly associated with catastrophic health expenditure *p* < 0.05. PLHIV in the racial/ethnic minority/migrants' group and those who earned less than ₦30,000 ($55) were statistically significantly associated with CHE at *p* < 0.001 with OR of 28.7 and 3.15, respectively.

**Conclusions:**

The study, therefore, highlights the widespread financial hardship faced by PLHIV in accessing healthcare, and the need for policies to increase financial risk protection.

**Abbreviations**
ARV: antiretroviral
CHE: catastrophic healthcare expenditure
LGBTQA+: lesbian, gay, bisexual, transgender, queer/questioning, asexual
OOP: out‐of‐pocket payment
OR: odds ratio
PLHIV: people living with HIV
UHC: Universal Health Coverage

## 1 INTRODUCTION

The high cost of healthcare has been known to pose a significant financial burden on individuals and households, particularly those residing in low‐ and middle‐income countries [1, 2]. Individuals with HIV and AIDS are not exempted due to their lifelong medication and treatment requirements, making healthcare costs even more challenging to bear. In many regions, healthcare costs create a barrier to accessing treatment, thereby preventing people living with HIV/AIDS from receiving the necessary care. For instance, as much as 70% of Nigerians rely on out‐of‐pocket (OOP) expenditures to finance their healthcare [1, 2]. These OOP payments become catastrophic when households sacrifice their basic needs to pay for healthcare expenses, leading to financial hardships [1].

The World Health Organization (WHO) has recently recommended that health expenditures should not exceed 10% of total household consumption or 25% of income [3]. However, over 150 million individuals are incurring catastrophic health expenditure (CHE), with more than 100 million sliding into poverty due to OOP payments for healthcare [3]. Interestingly, the focus on financial protection can help prevent households from experiencing financial hardships from healthcare expenses. In 2005, the Nigerian Federal Government launched the National Health Insurance Scheme to provide financial risk protection for Nigerians seeking healthcare, and in 2022, the National Health Insurance Authority Act, 2022 was signed into law to provide an oversight and regulatory function for Health Insurance Schemes in Nigeria [4, 5]. However, only a small percentage of Nigerians are health insured, with most being Federal Government workers [4]. HIV/AIDS is a global epidemic affecting over 84.2 million individuals, with approximately 40.1 million deaths recorded, and has been found to impose financial hardship on clients [6]. As of 2021, about 38.4 million individuals were living with HIV worldwide [6], with two‐thirds residing in sub‐Saharan Africa with Nigeria ranking fourth globally in terms of HIV burden [7, 8]. According to the Nigeria AIDS Indicator and Impact Survey, the prevalence of HIV in Nigeria is 1.4% (NAIIS 2022). Of the 1.4% infected with HIV, Akwa‐Ibom state (in the South‐south zone) has the highest prevalence of 5.6%, while Katsina state (in the North‐west zone) has the lowest prevalence of 0.3% with Lagos sitting with the highest prevalence in the South‐west with a prevalence of 1.3% [9].

Poverty and HIV/AIDS are interrelated, with poverty being a significant factor in HIV and AIDS transmission [10, 11]. HIV and AIDS can lead to poverty and may worsen existing poverty.

Developing countries like Nigeria have little or no social security systems for the poor and vulnerable individuals, making the financial impact of HIV more severe in Nigeria than in developed countries. The primary sources of finance for the health sector in Nigeria are the three tiers of government, public general revenue from various forms of taxation, health insurance institutions, the private sector, and donors, with out‐of‐pocket payments also contributing significantly. Studies show that households affected by HIV/AIDS have lower income levels than those without the disease, with increased health expenditures, loss of productivity, and asset sales associated with HIV‐affected households [11, 12, 13]. It has been proven that OOP expenses, comorbidities, low socioeconomic status, and transportation costs are significant contributors to CHE among HIV patients in Nigeria [14].

In light of the above‐identified findings combined with the existing studies on the economic burden of HIV and AIDS on households in some states in Nigeria, there is still a paucity of knowledge as regards the extent of financial hardship vis‐a‐vis. catastrophic health expenditure and the specific contributory factors to it among people living with HIV (PLHIV) in Lagos, hence the contribution of this study. It aimed to determine the CHE among PLHIV in Lagos and identify factors associated with CHE amongst them. Through this research, we aimed to deepen our understanding of the challenges faced by PLHIV and AIDS and to inform policy and practice in the provision of healthcare services.

## 2 METHODOLOGY

### 2.1 The setting, study design, and participants

The study was carried out in Lagos State, one of the Southwestern States in Nigeria, among participants drawn from 25 primary, secondary, and tertiary healthcare facilities and 5 one‐stop shops that provide HIV care and treatment services with the involvement of local community‐based organizations and networks of PLHIV. The one‐stop shop is a concept for providing nondiscriminatory HIV and psychosocial services to the KPs (LGBTQA+). It is of utmost importance to note that Lagos State has a prevalence of 1.3% of HIV among the population [15].

An observational, cross‐sectional study design was applied to investigate OOP and CHE among PLHIV and identify potential differences across the different subpopulations. The data were collected over 2 months among selected participants who had a confirmed HIV‐positive status. Participants were 18 years of age and older, living in and accessing care services in the city of Lagos. They must have accessed care or have been on treatment for at least 12 months and be able to respond to an interviewer‐administered questionnaire. The research assistants obtained the participants' informed consent before conducting the interview, and all participants gave their consent to the study.

### 2.2 Sample size determination

The minimum sample size was determined using the standard formula for descriptive studies [16] using a standard normal deviation of 1.96, and a *p* value of 0.5 for maximum variability, with a margin of error of 0.05. The minimum sample size calculated was 384. Giving allowances for a 20% nonresponse rate, the sample size was increased to 460. A total of 578 PLHIV, including members of the key populations (KPs) of clients receiving treatment in each of the different facilities across the state, were interviewed.

### 2.3 Sampling

Stratified simple random sampling was used to ensure proportionate representation and inclusion of all populations of interest based on their total size. This included KPs such as MSMs, CSWs, and transgender clients, as well as clients from health facilities. Within these groups, respondents were also selected proportionately by gender and age group, from a total of 25 high‐burden health facilities and 5 one‐stop‐shop facilities across Lagos.

### 2.4 Study instrument

The study instrument was an interviewer‐administered, pretested questionnaire developed from a literature review on the subject. The instrument had three sections: the first section dealt with the sociodemographic and economic characteristics of the respondents, the second section assessed the respondents' personal/household healthcare expenditure and expenditure made on transportation and feeding every month, and the third and fourth sections investigated the coping mechanisms of respondents with OOP payment for healthcare including health insurance enrollment. Face validation of the instrument was done by all the investigators, and the Cronbach's alpha reliability coefficient computed was 0.74.

### 2.5 Data collection technique

Five research assistants were trained to conduct face‐to‐face interviews at the selected health facilities. Individual survey participants were provided with the anonymity paper version of the questionnaire and interviewed by the research assistants who helped input information into the web‐based version of the questionnaire deployed via survey monkey developed especially for the study and compatible with all mobile platforms; this was to mitigate the challenges of digital survey for areas with limited connectivity or preference for the paper version.

### 2.6 Variables

Our explanatory variables included age, gender at birth, gender after birth, education completed, occupation, perception of the current state of health, and personal and household monthly income, while the outcome variable was the “presence or absence of CHE”. The “proportionality of income” approach was used to examine catastrophic expenditure [17]. A respondent was deemed to have incurred CHE if OOP expenditure on health as a fraction of the patient's personal or household annual income (less food expenditure) exceeded the specified threshold, which was set at 40% in this study [17]. The threshold of 40% was chosen because it has been widely used for several previous studies exploring catastrophic expenditure [17, 18]. To calculate the health costs, information was collected on hospital consultations, investigations, drugs (non‐ARV drugs since ARV drugs are free of charge), in‐patient hospital stays, and transportation costs directly related to hospital care. All costs related to the healthcare visits to the hospital premises were captured and added together. These were added to make up the total health expenditure incurred for health care. To estimate the annual household income, the monthly income was requested from all income earners within the household (cumulatively summed up as the income of the patient). An average was estimated for those respondents without a steady or formal source of income. Daily and weekly paid respondents were converted to monthly income using an appropriate multiplier (26 days or 4 weeks, respectively, to exclude Sundays). The annual income was calculated by multiplying the estimated monthly income by 12. At the time when the data were collected, the sum of N550 was equivalent to $1.

### 2.7 Data management

Completed questionnaires from the web platform were cleaned and coded on Microsoft Excel 2016 and exported to STATA SE 12 (STATA CORP LLC, College Station, TX, USA) where it was analyzed. Descriptive statistics including frequency tables were used to summarize the socioeconomic and health expenditure data as appropriate. Catastrophic health expenditure was constructed as “not catastrophic = 0”; “catastrophic = 1” for the bivariate analysis and the logistic regression. Associations between the explanatory variables such as age, gender, occupation, income, and so on and the outcome variable (categorized presence or absence of CHE) were investigated at the 5% level of significance. Logistic regression models that accounted for the survey design were fitted to identify the independent predictors of CHE. All significant variables in the bivariate analysis were fitted into the logistic regression model and presented as unadjusted odd ratios.

### 2.8 Ethical considerations

Ethical approval was obtained from the Health Research Ethics Committee of the Lagos State University Teaching Hospital Ikeja to carry out this study with approval number LREC/06/10/1390. An electronic/paper‐based consent was obtained from each respondent with the assurance of confidentiality of the information and their right to withdraw from the study at any point in time. Only the patient ID numbers of the sampled ART patients were used. The participants were made to understand that involvement was voluntary and the study posed no risk to them.

## 3 RESULTS

About 30.5% of respondents were less than 30 years old, 59% were females at birth, and close to 61% were females after birth. More than half (52.9%) were either unskilled workers or unemployed, and nearly 6% were sex workers. About 83% reported that they had excellent or very good health status, while approximately 35% earned ₦30,000 ($42.6) or less as personal monthly income, and the majority (92%) reported that they had no household monthly income. About 10% were homosexual, and a majority (70.2%) did not belong to any of the groups (Table 1).

**Table 1:** Sociodemographic characteristics of respondents.

Sociodemographic variable	*N* = 578
	Frequency (%)
**Age (mean ± SD)**	38.6 ± 12.0
<30	176 (30.5)
30–39	134 (23.2)
40–49	150 (26.0)
50–59	84 (14.5)
≥60	34 (5.9)
**Gender at birth**	
Female	342 (59.2)
Male	226 (39.1)
Prefer not to say	10 (1.7)
**Gender after birth**	
Female	351 (60.7)
Male	213 (36.9)
Transgender female	13 (2.3)
Transgender male	1 (0.2)
**Education completed**	
None	25 (4.3)
Primary	69 (11.9)
Secondary	272 (47.1)
Tertiary	164 (28.4)
Postgraduate	48 (8.3)
**Occupation**	
Unskilled/unemployed	306 (52.9)
Skilled	171 (29.6)
Professional	58 (10.0)
Sex worker	34 (5.9)
Clergy/retired	9 (1.6)
**Perception of current Health Status**	
Excellent/very good	480 (83.0)
Good	80 (13.8)
Poor/fair	18 (3.1)
**Number of people in your household**	
<5	352 (60.9)
≥5	226 (39.1)
**Personal monthly income (mean ± SD)**	29,880.69 ± 44,949.36
**Median (IQR)**	20,000 (0–40,000)
Nil	192 (33.3)
≤₦30,000	204 (35.4)
₦31,000–60,000	107 (18.5)
>₦60,000	74 (12.8)
**Household monthly income (mean ± SD)**	4616.98 ± 20,312.16
**Median (IQR)**	0 (0–0)
Nil	531 (92.0)
≤₦30,000	17 (3.0)
₦31,000–60,000	17 (3.0)
>₦60,000	12 (2.0)
**Group**	
Homosexual	58 (10.0)
Sex worker/a person who injects substances such as heroin	33 (5.7)
Bisexual	15 (2.6)
Racial/ethnic minority/migrant	54 (9.3)
None of the above	406 (70.2)
Prefer not to answer	12 (2.1)

The respondents incurred a mean consultation cost of N662 ($1.2) for outpatient and inpatient services over the past 12 months. The 5th–95th percentile ranged from N0 to N8000. The mean monthly expenditure was N29,282 ($53.2) on food and N8364 ($15.2) on healthcare. The 5th–95th percentile for monthly food spending was N4350 ($8) to N95,500 ($174). For monthly healthcare spending, the range was N0–N30,000 ($55) (Table 2).

**Table 2:** Respondents' expenditure on health, transport, and food.

Costs incurred in the last 12 months (outpatient and inpatient) (*N*)	5% percentile	Median	95%
Consultation fee (*n* = 416)	0	0	8000
Investigative cost (*n* = 415)	0	0	12,650
Non‐ARV drugs/supplements cost (*n* = 418)	0	0	12,300
Transportation fee (*n* = 451)	0	0	36,000
Food and accommodation (*n* = 445)	0	2000	240,000
Foregone income during treatment (*n* = 403)	0	0	2000
Estimated monthly amount household spend on Healthcare (*n* = 459)	0	5000	30,000
Estimated monthly amount household spend on Food (*n* = 459)	4350	30,000	95,500

Close to 60% of respondents experienced catastrophic healthcare expenditures, while about 40% did not. Approximately 30% of respondents borrowed money to pay for some of their treatments. Almost all (96%) had no health insurance. About 30.3% said that sometimes the cost of transportation made it difficult for them to get to their health clinic (Table 3).

**Table 3:** Healthcare expenditure among respondents.

Healthcare expenditure	Frequency (%)
**How did you cope with treatment cost**	
Borrowed money	172 (29.8)
Family assistance	168 (29.0)
Health insurance	11 (1.9)
Sold household assets	9 (1.6)
Used savings	218 (37.7)
**Enrolled for health insurance**	
Yes	23 (4.0)
No	555 (96.0)
**Last year, the cost of transportation made it difficult for you to get to your health clinic in the past 12 months**	
Almost always	63 (10.9)
Often	64 (11.1)
Sometimes	175 (30.3)
Seldom	18 (3.1)
Never	258 (44.6)
**Number of times you were unable to get medical care because you were unable to pay**	
Almost always	11 (1.9)
Often	8 (1.4)
Sometimes	51 (8.8)
Seldom	18 (3.1)
Never	490 (84.8)
Pays extra for medical care	23 (4.0)

Gender at birth, gender after birth, and occupation were found to be statistically significantly associated with catastrophic health expenditure among the respondents. Approximately 61% of those who were female at birth (vs. 49% males) and close to two‐thirds (63.7%) who were unskilled/unemployed respondents (vs. skilled [54%], professionals [50%]) experienced catastrophic healthcare expenditure, *p* < 0.05 (Table 4).

**Table 4:** Relationship between CHE and sociodemographic characteristics of respondents.

Sociodemographic variable	CHE (%)	Non‐CHE (%)	X^2^	*p*‐value
**Age**				
<30	92 (52.3)	84 (47.7)	3.4	0.486
30–39	75 (56.0)	59 (44.0)		
40–49	89 (59.3)	61 (40.7)		
50–59	53 (63.1)	31 (36.9)		
≥60	18 (52.9)	16 (47.1)		
**Gender at birth**				
Female	208 (60.8)	134 (39.2)	9.9	0.007*
Male	111 (49.1)	115 (50.9)		
Prefer not to say	8 (80.0)	2 (20.0)		
**Gender after birth**				
Female	215 (61.3)	136 (38.8)	18.7	<0.001*
Male	110 (51.6)	103 (48.4)		
Transgender female	1 (7.7)	12 (92.3)		
Transgender male	1 (100)	0 (0.0)		
**Education completed**				
None	13 (52.0)	12 (48.0)	2.8	0.586
Primary	36 (52.2)	33 (47.8)		
Secondary	152 (55.9)	120 (44.1)		
Tertiary	94 (57.3)	70 (42.7)		
Postgraduate	32 (66.7)	16 (33.3)		
**Occupation**				
Unskilled/unemployed	195 (63.7)	111 (36.3)	29.2	<0.001*
Skilled	93 (54.4)	78 (45.6)		
Professional	29 (50.0)	29 (50.0)		
Sex worker	6 (17.7)	28 (82.4)		
Retired/clergy	4 (44.4)	5 (55.6)		
**Perception of current Health Status**				
Excellent/very good	259 (54.0)	221 (46.0)	9.6	0.008*
Good	53 (66.3)	27 (33.8)		
Fair/poor	15 (83.3)	3 (16.7)		
**Number of people in your household**				
<5	182 (51.7)	170 (48.3)	8.7	0.003*
≥5	145 (64.2)	81 (35.8)		
**Personal monthly income (mean ± SD)**				
Nil	104 (54.2)	88 (45.8)	47.5	<0.001*
≤₦30,000	147 (72.1)	57 (27.9)		
₦31,000–60,000	56 (52.3)	51 (47.7)		
>₦60,000	20 (27.0)	54 (73.0)		
**Household monthly income (mean ± SD)**				
Nil	299 (56.3)	232 (43.7)	2.9	0.405
≤₦30,000	12 (70.6)	5 (29.4)		
₦31,000–60,000	11 (64.7)	6 (35.3)		
>₦60,000	5 (41.7)	7 (58.3)		
**Belong to these groups (currently or in the past)**				
Homosexual	11 (19.0)	47 (81.0)	81.7	<0.001*
Sex worker/a person who injects substances such as heroin	6 (18.2)	27 (81.8)		
Bisexual	6 (40.0)	9 (60.0)		
Racial/ethnic minority/migrant	48 (88.9)	6 (11.1)		
None of the above	248 (61.1)	158 (38.9)		
Prefer not to answer	8 (66.7)	4 (33.3)		

* 5% significance level.

Respondents whose personal income was less than or equal to ₦30,000 ($42.6) experienced high (72%) catastrophic healthcare expenditure as compared to those who earned higher (₦31,000–60,000; 52%, >₦60,000; 27%). Respondents' group, perception of current health status, and the number of people in their households were significantly associated with catastrophic health expenditure *p* < 0.05 (Table 4).

PLHIV in the racial/ethnic minority/migrant group and those who earned less than ₦30,000 ($55) were statistically significantly associated with catastrophic healthcare expenditure at *p* < 0.001 with OR 28.7 and 3.15, respectively (Table 5).

**Table 5:** CHE predictors among respondents.

CHE predictors	Adjusted OR	95% CI	*p*‐value
**Gender at birth**			
Prefer not to say	1		
Male	0.21	0.02–1.66	0.140
Female	0.24	0.03–1.69	0.153
**Gender after birth**			
Male	1		
Female	0.75	0.12–4.70	0.766
Transgender female/male	0.28	0.05–1.60	0.153
**Occupation**			
Unemployed/unskilled	1		
Clergy/retired	0.45	0.10–2.04	0.298
Sex worker	0.80	0.18–3.55	0.771
Skilled worker	0.96	0.61–1.51	0.869
Professional	0.54	0.29–1.05	0.069
**Perception of health status**			
Poor/fair	1		
Good	1.04	0.08–12.99	0.976
Excellent/very good	0.60	0.05–7.31	0.691
**Number of people living in a household**	1.10	0.74–1.64	0.624
**Personal monthly income**			
Nil	1		
<30k	3.15	1.97–5.02	<0.001*
31–60k	2.09	1.17–3.72	0.012*
>60k	0.90	0.45–1.82	0.771
**Groups**			
Sex worker/inject substance	1		
Bisexual	2.99	0.44–20.16	0.261
Homosexual	0.80	0.17–3.76	0.782
Racial/ethnic minority/migrant	28.71	5.25–157.09	<0.001*
None of the above	5.85	1.35–25.33	0.018*

* 5% significance level.

## 4 DISCUSSION

The delivery of the healthcare system is being faced with a lot of inequalities, with CHE serving as a fundamental challenge to it. Evaluating the incidence and intensity of HIV‐related CHE will help in providing insight into the UHC and also reflect the economic burden of HIV on patients and their families [17, 18].

There was a predominance of female respondents of 59.2% in this study, which is consistent with the outcome obtained from another study in Nigeria with 60% females. It was also found that the healthcare expenditure catastrophic was 57%, similar in percentage to what was reported in another study [17], but higher than what was observed in some other sub‐Saharan countries [19, 20, 21]. This is also higher than the CHE in the general Nigerian population [22]. The high catastrophic health expenditure is potentially a consequence of health system reforms not being actualized, along with limited aid for accessing healthcare services [20, 23]. There is a pressing need in Nigeria for innovative social safety net programs to help households manage routine healthcare needs and emergencies. The increased risk and vulnerability to incurring CHE as seen in this study have important policy implications and have also been brought to the fore in prior studies [19, 20, 23].

Another key aspect is healthcare insurance enrollment, which was very low (4%) in this study. This low value was similar to what was observed in other low‐ to medium‐income countries [17, 24]. This study found that health insurance status did not have a statistically significant association with catastrophic health expenditure among respondents, similar to the finding from a study in Iran [23]. This suggests that other factors, such as the cost of healthcare services, household income level, and higher expenses for transportation, food, and accommodation related to inpatient and outpatient care, may exert a greater influence on the risk of catastrophic spending on health. It is therefore critical that the government implement policies to minimize inequities in welfare provision across the nation.

Furthermore, this study revealed that nonmedical‐related costs like transportation fares, which are invariably greater for the poor living far from health facilities, food‐related costs, nonroutine tests, and inadequate care in primary health care facilities largely influence CHE, consistent with findings from other studies [20, 25]. Adherence to ART medications was again affected by the need for frequent visits, which led to CHE from increased transportation costs. Likewise, the higher use of patients' savings (37.7%) for healthcare in HIV services in this study was in agreement with what was found in a study where being a patient and a payer for medical bills increased susceptibility to the CHE. People with poor household incomes face more burdens of CHE, which is a hindrance to the attainment of UHC as more households of poor status are further pushed into poverty [26, 27, 28, 29]. Poverty is both a cause and a consequence of HIV/AIDS. Poverty increases vulnerability to HIV infection, while HIV/AIDS can lead to or exacerbate poverty. These findings highlighted the urgent need to recommend that policymakers increase public healthcare funding and implement social health protection programs to completely replace OOP health payments, especially among PLHIV. Such actions would provide financial risk protection, which is currently lacking for many households in Nigeria [27]. This study also observed that there was a statistically significant association between respondents' occupation, perception of health status, household size, income, and incurring CHE. The occupation of the household members may impact their access to health insurance and healthcare services. Furthermore, respondents who perceived their health status to be poor may face an increased risk of catastrophic health expenditure since their greater healthcare needs and expenses lead to higher healthcare costs. The size of the household may also affect the risk of catastrophic health expenditures, as larger households may have more healthcare expenses to cover. Moreover, the total salary earned by households can impact their capacity to pay for healthcare expenses, since higher incomes may offer greater financial flexibility and the ability to allocate resources.

Overall, these findings suggest that multiple factors can influence the risk of catastrophic health expenditure and that policies and interventions aimed at reducing this risk may need to consider a range of economic and social factors beyond health insurance coverage alone.

The study focused only on a specific geographic region (South Western Nigeria) and a specific population (people living with HIV/AIDS), which may limit the generalizability of the findings to other regions or populations. The study used a cross‐sectional design, which means that it was conducted at a single point in time and cannot establish causality or examine changes over time.

## 5 CONCLUSION

This study found that a significant proportion of PLHIV in South West Nigeria incurs CHE, which can have severe consequences for their health and well‐being. Also, households with lower economic status, poorer perception of current health status, larger household sizes, and lower salaries were at higher risk of experiencing CHE. Also, we found a very low health insurance uptake among respondents, even though their health insurance status did not have a statistically significant effect on the likelihood of experiencing CHE.

Our recommendations include expanding access to responsive health insurance, extending funding for HIV support programs, especially for transportation, feeding, and accommodation during the clinic visits of PLHIV, improving economic opportunities for households, increasing public awareness of HIV/AIDS and its treatment, and conducting further research to better understand the drivers of CHE among PLHIV.

## AUTHOR CONTRIBUTIONS

**Adeyinka Adeniran**: Conceptualization (lead); Data curation (equal); Formal analysis (equal); Investigation (equal); Methodology (lead); Project administration (lead); Supervision (equal); Validation (lead); Writing—original draft (equal); Writing—review & editing (equal). **Omobola Y. Ojo**: Methodology (equal); Writing—original draft (equal); Writing—review & editing (equal). **Florence C. Chieme**: Data curation (equal); Formal analysis (lead); Visualization (equal); Writing—review & editing (equal). **Yeside Shogbamimu**: Conceptualization (equal); Investigation (lead); Methodology (equal); Project administration (equal); Resources (equal); Supervision (lead); Writing—review & editing (equal). **Helen O. Olowofeso**: Conceptualization (equal); Funding acquisition (lead); Investigation (equal); Methodology (equal); Project administration (lead); Resources (lead); Supervision (lead); Writing—review & editing (equal). **Imane Sidibé**: Conceptualization (equal); Funding acquisition (lead); Investigation (equal); Resources (lead); Writing—review & editing (equal). **Oladipupo Fisher**: Funding acquisition (equal); Investigation (equal); Resources (equal); Writing—review & editing (equal). **Monsurat Adeleke**: Funding acquisition (equal); Investigation (equal); Project administration (equal); Resources (equal); Supervision (equal).

## CONFLICT OF INTEREST

The authors declare no conflicts of interest.

## ETHICS STATEMENT

The study protocol was approved by the Health Research Ethics Committee of the Lagos State University Teaching Hospital Ikeja with approval number LREC/06/10/1390.

## INFORMED CONSENT

An electronic/paper‐based written consent was obtained from each respondent with the assurance of confidentiality of the information and their right to withdraw from the study at any point in time.

## Data Availability

Data are available upon request to the authors. The data are not publicly available due to privacy or ethical restrictions.
